# Chronic C-Type Natriuretic Peptide Infusion Attenuates Angiotensin II-Induced Myocardial Superoxide Production and Cardiac Remodeling

**DOI:** 10.1155/2012/246058

**Published:** 2012-07-17

**Authors:** Yasuhiro Izumiya, Satoshi Araki, Hiroki Usuku, Taku Rokutanda, Shinsuke Hanatani, Hisao Ogawa

**Affiliations:** Department of Cardiovascular Medicine, Graduate School of Medical Sciences, Kumamoto University, Kumamoto 860-8556, Japan

## Abstract

Myocardial oxidative stress and inflammation are key mechanisms in cardiovascular remodeling. C-type natriuretic peptide (CNP) is an endothelium-derived cardioprotective factor, although its effect on cardiac superoxide generation has not been investigated in vivo. This study tested the hypothesis that suppression of superoxide production contributes to the cardioprotective action of CNP. Angiotensin II (Ang II) or saline was continuously infused subcutaneously into mice using an osmotic minipump. Simultaneously with the initiation of Ang II treatment, mice were infused with CNP (0.05 **μ**g/kg/min) or vehicle for 2 weeks. The heart weight to tibial length ratio was significantly increased by Ang II in vehicle-treated mice. Treatment with CNP decreased Ang II-induced cardiac hypertrophy without affecting systolic blood pressure. Echocardiography showed that CNP attenuated Ang II-induced increase in wall thickness, left ventricular dilatation, and decrease in fractional shortening. CNP reduced Ang II-induced increases in cardiomyocyte size and interstitial fibrosis and suppressed hypertrophic- and fibrosis-related gene expression. Finally, CNP decreased Ang II-induced cardiac superoxide production. These changes were accompanied by suppression of NOX4 gene expression. Our data indicate that treatment with CNP attenuated Ang II-induced cardiac hypertrophy, fibrosis, and contractile dysfunction which were accompanied by reduced cardiac superoxide production.

## 1. Introduction

C-type natriuretic peptide (CNP) belongs to the natriuretic peptides (NPs) family, which was originally isolated from porcine brain tissues and later established as a positive regulator of endochondral ossification [1]. In the cardiovascular system, CNP is secreted from endothelial cells and cardiac fibroblasts, and acts as an autocrine/paracrine regulator [2, 3]. Myocardial production and circulating CNP levels are increased in patients with chronic heart failure [4, 5], suggesting that CNP has an important role in the pathogenesis of heart failure. ANP and BNP bind to natriuretic peptide receptor (NPR)-A, inducing natriuresis and diuresis, whereas CNP preferentially binds to its specific receptor, NPR-B. Because of the relative abundance of NPR-B over NPR-A in cardiac fibroblasts and in cardiomyocytes, CNP reportedly has more potent antihypertrophic and antifibrotic properties, despite less hypotensive and less natriuretic effects compared with ANP and BNP [6, 7]. The recently designed novel compound CD-NP, which is a chimera of CNP and DNP, is a promising candidate for new-generation therapeutics using NPs [8]. It is therefore of interest to elucidate the precise role of CNP in cardiovascular pathophysiology from both a basic and clinical medicine perspective.

Previous experimental studies showed that long-term administration of CNP attenuates cardiac remodeling and maintains systolic function in murine models of myocardial infarction and myocarditis, independent of its hypotensive effect [9, 10]. Cardiomyocyte-specific overexpression of CNP attenuated cardiac hypertrophy in a murine model of myocardial infarction [11]. Conversely, transgenic rats with overexpression of a dominant-negative mutant of NPR-B showed exacerbated cardiac hypertrophy [12]. In vitro, it has been shown that CNP prevents agonist-induced cardiomyocyte hypertrophy via a cyclic GMP-dependent protein kinase-mediated mechanism [7, 13]. CNP also inhibits fibroblast proliferation and extracellular matrix production of cardiac fibroblasts more potently than ANP and BNP [6]. These data indicate that CNP acts directly on myocardial cells to protect against pathological stimuli.

Experimental and clinical evidence implicates a central role for angiotensin II (Ang II) in all stages of the cardiovascular continuum [14, 15], and the detrimental effects of Ang II are mediated, at least in part, by reactive oxygen species (ROS) [16–18]. To this end, the antioxidant activity contributes to the reported cardioprotective effects of ANP [19]. However, little is known about the effects of CNP on Ang II-induced cardiac ROS generation. Here, we investigated whether chronic CNP infusion could attenuates myocardial superoxide production in a mouse model of cardiac hypertrophy and remodeling induced by Ang II.

## 2. Materials and Methods

### 2.1. Animals

Ten-week-old male C57BL/6 mice were used in the present study. Ang II (Peptide Institute) or saline alone was subcutaneously infused via an osmotic minipump (Alzet model 1002) at the rate of 3.2 mg/kg/day for 2 weeks [20]. Simultaneously with the initiation of Ang II treatment, mice were infused with CNP (Peptide Institute, 0.05 *μ*g/kg/min) or vehicle by continuous subcutaneous infusion for 2 weeks. Systolic blood pressure (SBP) and heart rate (HR) of the conscious mice were monitored by the tail-cuff method (MK-2000ST; Muromachi). Transthoracic echocardiography was performed 2 weeks after the Ang II infusions. Mice were then euthanized and the hearts were weighed and harvested for further analysis. The serum level of CNP was measured using the CNP-22 EIA Kit (Phoenix Pharmaceuticals, Karlsruhe, Germany) according to the manufacturer's protocol [21]. All procedures were performed in accordance with the Kumamoto University animal care guidelines, which conformed to the Guide for the Care and Use of Laboratory Animals published by the US National Institutes of Health (NIH Publication No. 85-23, revised 1996).

### 2.2. Echocardiography

Transthoracic ultrasound cardiogram (UCG) was performed using a Xario system (Toshiba, Tokyo, Japan) equipped with a 12-MHz linear array transducer. M-mode images were recorded from the short-axis view at the high papillary muscle level. Left ventricular end-diastolic dimension (LVDd), end-systolic dimension (LVDs), intraventricular septum (IVS), and posterior wall thickness (PW) were measured. Fractional shortening (%FS) was calculated using the following equation: %FS = (LVDd − LVDs)/LVDd × 100. All recordings were performed in conscious mice. All echocardiography was performed by investigators who were blinded to the identity of the treatment group.

### 2.3. Real-Time PCR

Total RNA was prepared using a Qiagen RNeasy fibrous minikit, using the protocol supplied by the manufacturer, and cDNA was produced using the ThermoScript RT-PCR Systems (Invitrogen, Carlsbad, CA). Real-time PCR was performed as described previously [22]. Transcript expression levels were determined as the number of transcripts relative to those for glyceraldehyde-3-phosphate dehydrogenase (GAPDH), and normalized to the mean value from control hearts.  Table 1 lists the primer sequences used in this study.

### 2.4. Histological Analysis

Heart sections were prepared and stained with hematoxylin and eosin (H&E) for overall morphology or Masson's trichrome (MT) to assess myocardial interstitial fibrosis [17]. At least 10 fields were selected randomly to determine myofiber size and myocardial interstitial fibrosis using Image *J* image analysis software, as described previously [17]. Dihydroethidium (DHE) was used to evaluate superoxide levels of cardiac tissue as described in detail previously [23]. DHE fluorescence of cardiac sections was quantified using Lumina Vision version 2.2 analysis software.

### 2.5. Statistical Analysis

All data are presented as mean ± SEM. Comparisons among groups were made by one-way ANOVA with Fisher's PLSD test. A level of *P* < 0.05 was accepted as statistically significant.

## 3. Results

### 3.1. Effects of Chronic CNP Infusion on Hemodynamic Parameters

To examine the effect of exogenous CNP supplementation on Ang II-induced cardiac remodeling, Ang II- and saline-infused mice were treated with CNP or vehicle for 2 weeks (Table 2). Ang II infusion for 2 weeks significantly increased SBP compared with the saline-infused control group (136 ± 6 versus 97 ± 4 mmHg, resp.). Circulating CNP levels were significantly increased in CNP-treated mice compared with vehicle-treated controls. The dose of CNP used (0.05 *μ*g/kg/min) had no additional effect on SBP in either Ang II- or saline-infused mice. HR was unaffected by Ang II infusion in both CNP- and vehicle-treated mice compared to saline-infused mice.

### 3.2. CNP Administration Attenuates Ang II-Induced Cardiac Dysfunction

**Figure 1:**
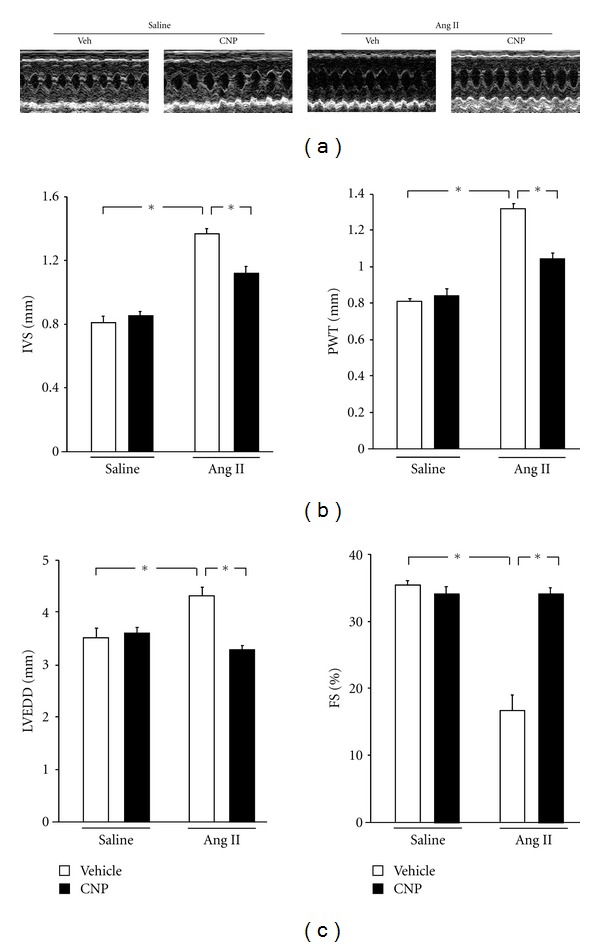
Effects of CNP on echocardiographic measurements. (a) Representative M-mode echocardiogram of vehicle- and CNP-treated mice 2 weeks after saline or Ang II infusion. (b) Interventricular septum (IVS) and posterior wall thickness (PWT) in vehicle- and CNP-treated mice 2 weeks after saline or Ang II infusion. (c) Left ventricular end-diastolic dimension (LVEDD) and fractional shortening (%FS) in vehicle- and CNP-treated mice 2 weeks after saline or Ang II infusion. Results are mean ± SEM (*n* = 3–6 each). *P < 0.05.

Figure 1(a) shows representative LV M-mode echocardiographic recordings. As shown in Figure 1(b), IVS and PWT were significantly increased by Ang II infusion in vehicle-treated mice, and these changes were suppressed by CNP treatment (without affecting SBP, as stated). Echocardiographic analysis also revealed significantly increased LVEDD and %FS in vehicle-treated mice 2 weeks after Ang II infusion (Figure 1(c)); however, these effects were significantly attenuated by CNP treatment. In contrast, long-term infusion of CNP had no effect on echocardiographic parameters in saline-infused mice.

### 3.3. CNP Infusion Attenuates Ang II-Induced Cardiomyocyte Hypertrophy

**Figure 2:**
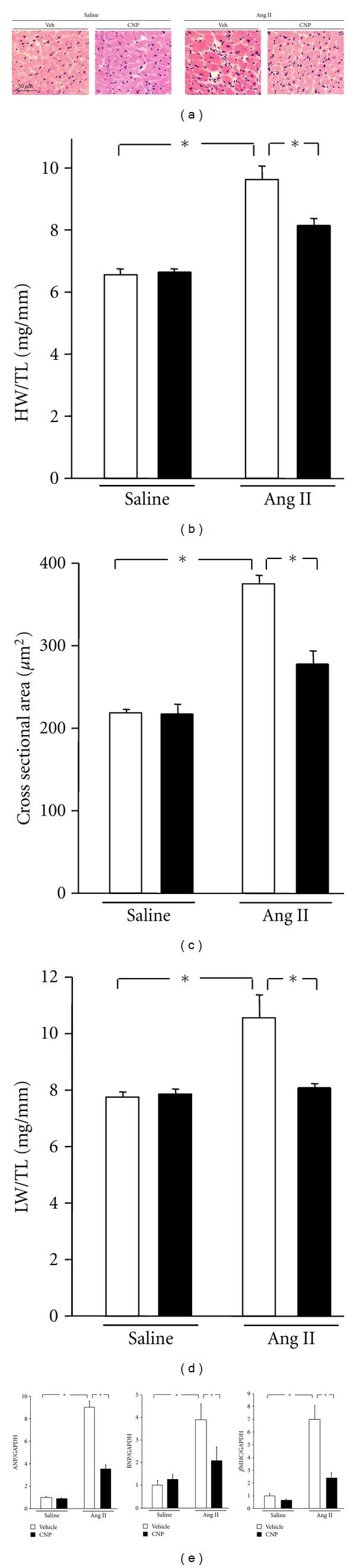
Effects of CNP on Ang II-induced cardiomyocyte hypertrophy. (a) Representative images of hematoxylin-eosin- (H&E-) stained heart sections. Scale bars: 50 *μ*m. (b) Heart weight to tibial length ratio (HW/TL) in vehicle- and CNP-treated mice 2 weeks after saline or Ang II infusion. (c) Quantitative analysis of cardiac myocyte cross-sectional area in vehicle- and CNP-treated mice 2 weeks after saline or Ang II infusion. Data were obtained from analysis of H&E-stained heart sections. (d) Lung weight to tibial length ratio (LW/TL) in vehicle- and CNP-treated mice 2 weeks after saline or Ang II infusion. (e) Atrial natriuretic peptide (ANP), B-type natriuretic peptide (BNP), and *β*-myosin heavy chain (*β*MHC) transcript expression in vehicle- and CNP-treated mice 2 weeks after saline or Ang II infusion. Results are mean ± SEM (*n* = 4–9 each). *P < 0.05.

Heart weight to tibial length (HW/TL) ratios were significantly increased by Ang II infusion in vehicle-treated mice (9.6 ± 0.5 versus 6.5 ± 0.2 mg/mm; Figure 2(b)), while treatment with CNP decreased this Ang II-induced cardiac hypertrophy by 16%. CNP had no effect on heart size in the saline-infused mice. Analysis of myocyte cross-sectional area in histological sections corroborated these findings (Figure 2(a)); the calculated area of myocytes was increased in vehicle-treated mice that underwent Ang II infusion, but this increase was largely blocked in mice given CNP (276 ± 16 versus 375 ± 11 *μ*m^2^; Figure 2(c)). CNP treatment of vehicle-treated mice had no effect on myocyte cross-sectional area. Consistent with this observation, Ang II infusion increased expression of the fetal-type cardiac genes ANP, BNP, and *β*-myosin heavy chain, and these upregulations were blocked by CNP treatment (Figure 2(e)). Two weeks after Ang II infusion, the lungs wet weight to TL ratios (LW/TL), a parameter used to assess pulmonary congestion, were significantly decreased in CNP-treated mice compared with vehicle-treated controls (8.1 ± 0.1 versus 10.6 ± 0.8 mg/g; Figure 2(d)).

### 3.4. CNP Infusion Reduces Ang II-Induced Myocardial Interstitial Fibrosis

**Figure 3:**
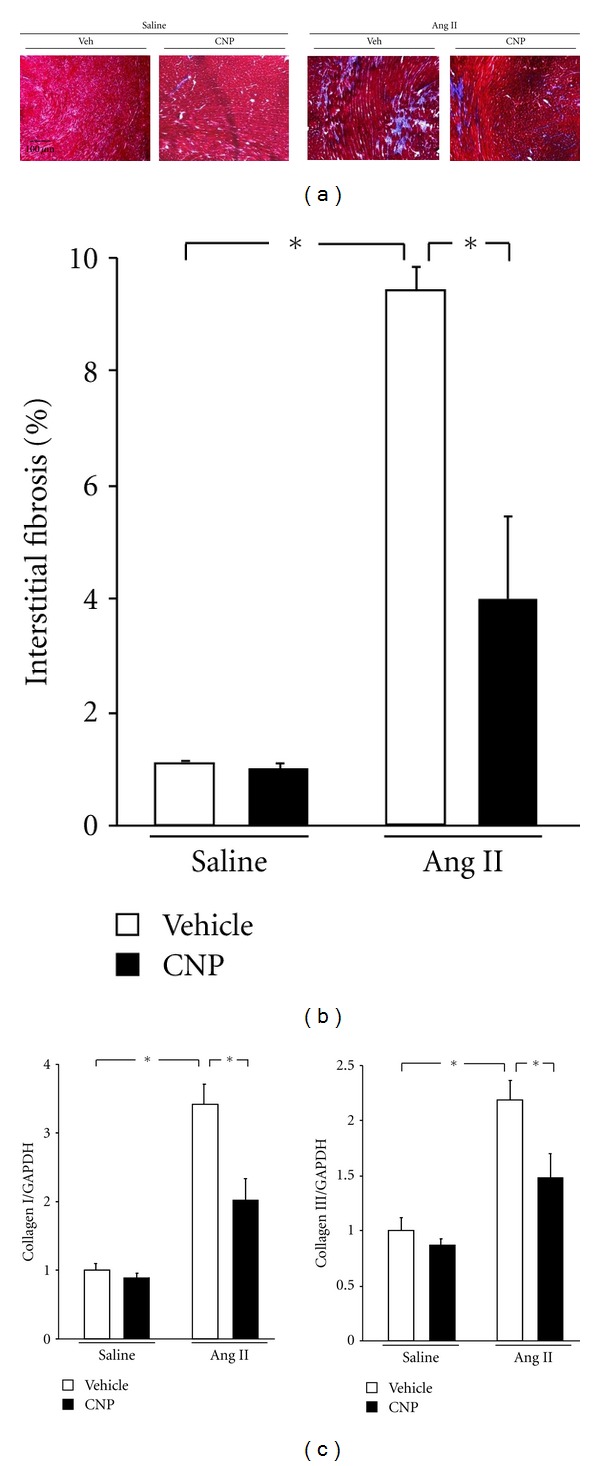
Effects of CNP on Ang II-induced myocardial interstitial fibrosis. (a) Representative images of Masson's trichrome (MT) staining of heart sections. Scale bars: 100 *μ*m. (b) Quantitative analysis of myocardial interstitial fibrosis in vehicle- and CNP-treated mice 2 weeks after saline or Ang II infusion. (c) Collagen I and III transcript expression in vehicle- and CNP-treated mice 2 weeks after saline or Ang II infusion. Results are mean ± SEM (*n* = 4–9 each). *P < 0.05.

To investigate the consequence of exogenous CNP administration on cardiac remodeling, we evaluated myocardial interstitial fibrosis (Figure 3(a)). Interstitial fibrosis was significantly increased in vehicle-treated mice 2 weeks after Ang II infusion (Figure 3(b)). However, treatment with CNP caused a significant decrease in this measure (4.0 ± 0.8 versus 9.4 ± 0.4%). In agreement with this observation, Ang II-induced gene upregulations of collagen I and III were significantly decreased by exogenous CNP treatment (Figure 3(c)). CNP infusion did not detectably affect fibrosis or collagen expression in saline-infused mice.

### 3.5. CNP Supplementation Diminishes ROS Production in Hearts Subjected to Ang II Infusion

**Figure 4:**
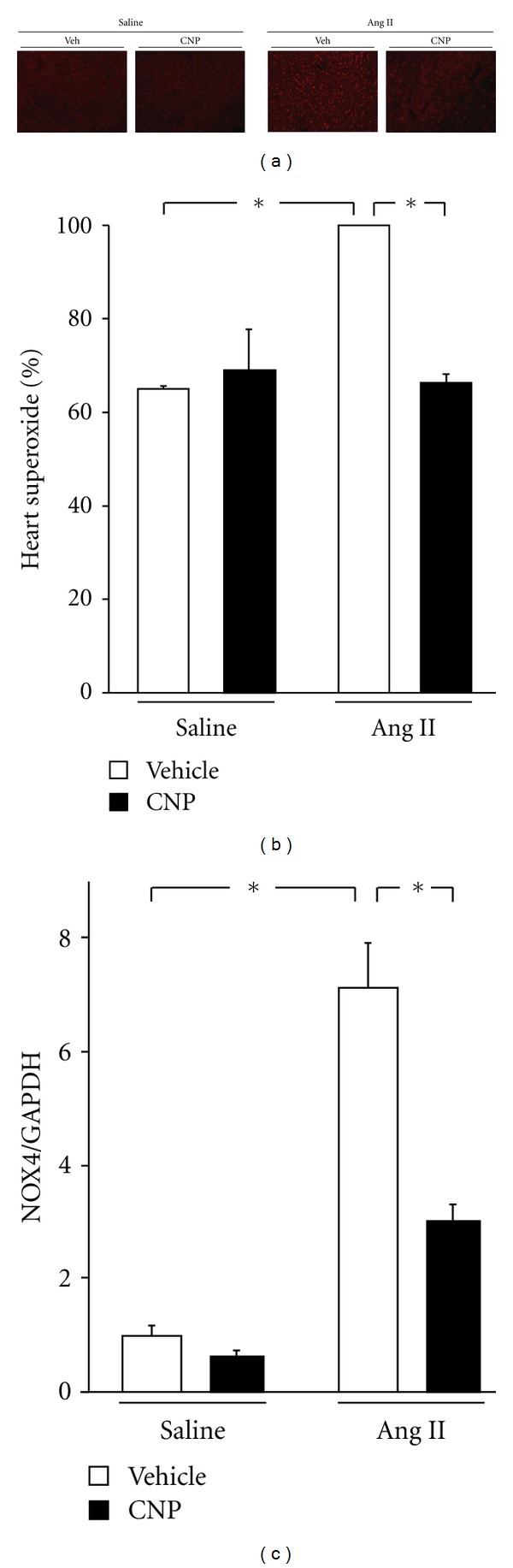
Effects of CNP on Ang II-induced ROS production. (a) Representative photomicrograph of myocardium stained with DHE. (b) DHE fluorescence was quantified to evaluate superoxide levels in situ and expressed relative to values obtained for vehicle-treated with Ang II infusion group (as 100%). (c) NOX4 transcript expression in vehicle- and CNP-treated mice 2 weeks after saline or Ang II infusion. Results are mean ± SEM (*n* = 4–9 each). *P < 0.05.

Myocardial superoxide generation is a key mechanism underlying the Ang II-induced cardiac remodeling [24]. We therefore investigated whether CNP administration could attenuates Ang II-induced superoxide production in mice heart. Myocardial tissue sections were stained with DHE to determine superoxide production in vehicle- and CNP-treated mice 2 weeks after Ang II infusion (Figure 4(a)). Ang II infusion increased superoxide generation in vehicle-treated mice by a factor of 1.5, but treatment with CNP markedly diminished Ang II-induced cardiac superoxide production (Figure 4(b)). Furthermore, Ang II induced the gene upregulation of NOX4, an NADPH oxidase subunit, and this was clearly suppressed by treatment with CNP (Figure 4(c)). In contrast, CNP treatment of vehicle-treated mice had no effect on myocardial superoxide production or NOX4 gene expression. Transcript expression levels of other NADPH oxidase subunits such as p22 and p47^phox^ were not changed by CNP treatment.

## 4. Discussion

This study provides the first in vivo evidence that continuous infusion of exogenous CNP attenuated Ang II-induced myocardial superoxide production and cardiac dysfunction. Administration of CNP significantly prevented Ang II-induced cardiac hypertrophy and LV dilatation, thereby maintaining myocardial contractile function without affecting SBP. CNP reduced Ang II-induced increases in cardiomyocyte size and interstitial fibrosis, and markedly suppressed hypertrophic and fibrotic gene expression. These beneficial effects were accompanied by reduced superoxide production and NOX4 gene expression in the Ang II-infused myocardium. Together, these findings suggested that CNP exerts antioxidant activity under conditions that promote cardiac hypertrophy and fibrosis, as a result, maintaining left ventricular systolic function.

 Cardiac hypertrophy is initially an adaptive response designed to maintain cardiac function in response to hemodynamic or neurohumoral stress. However, a prolonged external load leads to decompensated cardiac hypertrophy characterized by ventricular dilation and loss of contractile function. Although the role of ANP and BNP in cardiac hypertrophy and remodeling has been investigated in several in vitro and in vivo studies, limited data are available on the role of CNP in this process [3]. We used an Ang II infusion model in this study because multiple lines of evidence established that Ang II induces not only hypertension, but also directly contributes to cardiac remodeling [15]. Contractile function following Ang II infusion to mice varies among report. For example, Essick et al. reported that contractile function was not impaired by same dose (3.2 mg/kg/day) and same duration (2 weeks) of Ang II infusion as our present study [25]. On the other hands, Fujita et al. reported that lower dose of Ang II infusion (1.2 mg/kg/day) for 2 weeks reduced contractile function [26]. The reason for the discrepancy was probably due to different reagents, equipments, or mice strain.

Ang II-induced ROS generation is recognized as a key mechanism regulating cardiovascular remodeling [24]. With regard to the cellular source of ROS, Ang II-induced upregulation of NADPH oxidase subunits has been documented in various cardiovascular cell types including cardiomyocytes, fibroblasts, and vascular smooth muscle cells (VSMC) [27–29]. One of these subunits, NOX4, was implicated in Ang II infusion and/or pressure overload-induced myocardial hypertrophy [30–32]. This study revealed that NOX4 gene upregulation was dramatically suppressed by CNP treatment. It has been demonstrated that CNP prevents agonist-induced cardiomyocyte hypertrophy via a cyclic GMP-dependent protein kinase-mediated mechanism in vitro [7, 13]. Thus, we speculate that the antihypertrophic effect of chronic CNP infusion observed in this study was mediated by cyclic GMP-dependent manner and inhibition of NOX4 production.

 Another natriuretic peptide, ANP, can suppress ROS production in the cardiovascular system. For example, the antihypertrophic actions of ANP in vitro were accompanied by reduced levels of superoxide, mediated via the NPR-A/cGMP pathway [19]. We previously demonstrated that intravenous infusion of carperitide, the human form of ANP, significantly decreased serum ROS levels in patients with chronic heart failure and exogenous ANP exerted direct antioxidant properties in cardiomyocytes in vitro [33]. In contrast to ANP and BNP, CNP binds to and activates its specific receptor, NPR-B [3]. Both NPR-A and NPR-B activate cGMP-dependent signaling upon binding of their specific ligands, suggesting that the antioxidative properties of CNP were mediated via the NPR-B/cGMP pathway. In addition, all natriuretic peptides bind to NPR-C. Recent reports indicated that stimulation of NPR-C decreases NADPH oxidase activity and production of superoxide by attenuating the expressions of NOX4 and p47^phox^ subunits of NADPH oxidase in VSMC from spontaneous hypertensive rats [34]. We speculate that CNP inhibit the NOX4 expression by same mechanism in the present experiments.

 The synthetic reagents for ANP and BNP are currently used in a clinical setting with reported antiremodeling effects [35]. However, due to their prompt vasodilatation and natriuretic actions, these agents cannot be used in patients with severe hypotension as sometimes seen in acute heart failure. Compared to ANP and BNP, CNP exerts lower hypotensive and natriuretic effects, and more potent antihypertrophic and antifibrotic properties [36]. These functional differences accounted for the relative abundance of NPR-B over NPR-A in cardiovascular tissue [6, 7]. The reduced hypotensive action of CNP also makes it usable in hemodynamically unstable patients. Furthermore, these hemodynamic and antiremodeling aspects are ideal for long-term administration, because cardiac remodeling is generally developed as a result of long-term external stimulus. In this study, chronic CNP infusion had little effect on SBP; however, Ang II-induced cardiac remodeling was markedly prevented. Our results thus support the notion that long-term CNP administration is potentially useful in anti-remodeling strategies for chronic heart diseases [10].

 In conclusion, our data indicated that CNP protects against the development of Ang II-induced cardiac remodeling which was accompanied by reduced cardiac superoxide production. This novel mechanism of action makes CNP a potential additional anti-remodeling agent.

## Figures and Tables

**Table 1:** Primer sequences used for quantitative real-time PCR.

Gene		Primer sequence
ANP	Forward	5′-GAGAGACGGCAGTGCTTCTAGGC-3′
	Reverse	5′-CGTGACACACCACAAGGGCTTAGG-3′
BNP	Forward	5′-GGAAGTCCTAGCCAGTCTCC-3′
	Reverse	5′-TTGGTCCTTCAAGAGCTGTC-3′
*α*-MHC	Forward	5′-GACGAGGCAGAGCAGATCGC-3′
	Reverse	5′-GGGCTTCACAGGCATCCTTAGGG-3′
Collagen I	Forward	5′-GTCCCAACCCCCAAAGAC-3′
	Reverse	5′-CATCTTCTGAGTTTGGTGATACGT-3′
Collagen III	Forward	5′-TGGTTTCTTCTCACCCTTCTTC-3′
	Reverse	5′-TGCATCCCAATTCATCTACGT-3′
NOX-4	Forward	5′-TGGGCCTAGGATTGTGTTTA-3′
	Reverse	5′-CTGCTAGGGACCTTCTGTGA-3′
GAPDH	Forward	5′-TCACCACCATGGAGAAGGC-3′
	Reverse	5′-GCTAAGCAGTTGGTGGTGCA-3′

**Table 2:** Body weight, blood pressure, heart rate, and serum CNP concentration in vehicle-and CNP-treated mice at 2 weeks after Ang II infusion.

	Saline		Ang II	
	Vehicle	CNP	Vehicle	CNP
Body weight (g)	24 ± 0.3	25 ± 2.1	21 ± 0.4*	22 ± 0.6*
SBP (mmHg)	97 ± 4	87 ± 3	136 ± 6*	130 ± 7*
HR (bpm)	690 ± 16	693 ± 14	706 ± 8	699 ± 14
CNP (ng/mL)	0.559 ± 0.093	0.783 ± 0.050*	0.510 ± 0.066	0.701 ± 0.060*

Data are mean ± SEM (*n* = 4 − 9 mice per experimental group).

*P < 0.05 versus saline-infused and vehicle-treated mice.
